# β-diketone accumulation in response to drought stress is weakened in modern bread wheat varieties (*Triticum aestivum* L.)

**DOI:** 10.3389/fpls.2024.1401135

**Published:** 2024-08-09

**Authors:** Aswini Kuruparan, Peng Gao, Raju Soolanayakanahally, Santosh Kumar, Eliana Gonzales-Vigil

**Affiliations:** ^1^ Department of Cell and Systems Biology, University of Toronto, Toronto, ON, Canada; ^2^ Department of Biological Sciences, University of Toronto Scarborough, Toronto, ON, Canada; ^3^ Saskatoon Research and Development Centre, Agriculture and Agri-Food Canada, Saskatoon, SK, Canada; ^4^ Brandon Research and Development Centre, Agriculture and Agri-Food Canada, Brandon, MB, Canada

**Keywords:** cuticular waxes, drought, wheat, β-diketones, wax biosynthesis

## Abstract

Cuticular waxes coating leaf surfaces can help plants tolerate drought events by reducing non-stomatal water loss. Despite their role in drought tolerance, little is known about how cuticular wax composition has changed during breeding in Canadian bread wheat (*Triticum aestivum* L.) varieties. To fill in this gap, flag leaves of the Canadian Heritage Bread Wheat Panel, which include 30 varieties released between 1842 and 2018, were surveyed to determine if and how cuticular wax composition in wheat has changed at two breeding ecozones over this period. Following this, a subset of varieties was subjected to drought conditions to compare their responses. As expected, modern varieties outperformed old varieties with a significantly larger head length and reaching maturity earlier. Yet, when challenged with drought, old varieties were able to significantly increase the accumulation of β-diketones to a higher extent than modern varieties. Furthermore, RNAseq was performed on the flag leaf of four modern varieties to identify potential markers that could be used for selection of higher accumulation of cuticular waxes. This analysis revealed that the *W1* locus is a good candidate for selecting higher accumulation of β-diketones. These findings indicate that the variation in cuticular waxes upon drought could be further incorporated in breeding of future bread wheat varieties.

## 1 Introduction

Wheat is the most widely grown crop in the world accounting for 20% of the global population’s calorie and protein intake (Shiferaw et al., 2013; Bi et al., 2017). Nearly 90% of all wheat grown is the bread wheat variety (*Triticum aestivum* L.), with Canada being one of the largest producers holding a 10% share in the global wheat market (He et al., 2013; Jing et al., 2021). Most production occurs within Western Canada with Canadian Western Red Spring (CWRS) accounting for 60% of all wheat grown in the region (Kumar et al., 2019). However, global warming has led to fluctuations in soil moisture, with the frequency, duration, and severity of drought events rising globally from 2001 to 2020 (Dai, 2011; Chen et al., 2022). Climate models have predicted that Western Canada, one of the largest producers of wheat in the world, will experience an increase in the frequency and intensity of drought events posing a risk to productivity. Crops in Southern Alberta and Saskatchewan are often affected by drought, and historical trends reveal that low yields in bread wheat coincide with severe drought events (McCaig and DePauw, 1995). Similarly, projections indicate there is over an 80% chance that wheat production will fall below average under exceptional drought, especially in Canada and the United States (Leng and Hall, 2019). Clearly, there is an exceptionally high demand for drought-tolerant wheat varieties that can thrive in future climate scenarios. One mechanism that limits water loss is via cuticular wax accumulation on the leaf surface. Cuticular waxes can reduce non-stomatal water loss under drought settings and also assist in reflecting UV radiation, which can lower leaf transpiration and canopy temperature, without reducing stomatal conductance (Laila et al., 2017; Willick et al., 2018).

These properties are conferred by the hydrophobicity of the components that make up the cuticular waxes. Waxes are predominantly composed of aliphatic compounds, derived from very long-chain fatty acids (VLCFAs, more than 18 carbons long) synthesized at the endoplasmic reticulum surface by the fatty acid elongation complex (Yeats and Rose, 2013). In bread wheat, VLCFAs can undergo several modifications giving rise to homologous series of compounds through three biosynthetic pathways: the alkane-forming pathway (responsible for alkanes, secondary alcohols, and ketones), the alcohol-forming pathway (responsible for primary alcohols and wax esters), and the diketone-forming pathway (responsible for β-diketones, hydroxy-β-diketones, and 2-alkanols) (Tulloch and Hoffman, 1973; Hen-Avivi et al., 2016; Sun et al., 2023). Several genes involved in the biosynthesis of cuticular waxes in wheat have already been characterized. Decarbonylation of VLCFAs to odd-chain alkanes is carried out by the combined activity of CER1 and CER3, with two homologs characterized in wheat (*TaCER1–1A* and *TaCER1–6A*) (Li et al., 2019; He et al., 2022). Reduction to primary alcohols is catalyzed by the fatty acyl-CoA reductase (*FAR*), with five genes in wheat implicated in the biosynthesis of primary alcohols in the leaf (*TaFAR1* through *5*). Heterologous expression of *TaFAR*s in yeast has shown that these enzymes have preference for making alcohols of specific lengths (Wang et al., 2015a, 2016). More recently, it was discovered that diketone production is controlled by a biosynthetic gene cluster referred to as the *WAX1 (W1)* locus. The W1 gene cluster is present in the B and D subgenomes and composed of a thioesterase (*DMH*), a type-III polyketide synthase (*DKP*), and a cytochrome P450 oxidase (*DMC*); and the long non-coding RNA encoded by *INHIBITOR of WAX1* (*Iw1*) (Hen-Avivi et al., 2016; Huang et al., 2017; Sun et al., 2023).

Given the protective role of cuticular waxes against drought, the chemical composition can change to better meet function. This is particularly seen with the accumulation of β-diketones and alkanes. The abaxial surface of wheat leaves, flower heads and stems display a bluish-white phenotype referred to as glaucousness imparted by β-diketone deposition (Bi et al., 2017; Su et al., 2020). Under drought-stressed field conditions, glaucousness can reduce leaf temperatures by 0.7°C (Richards et al., 1986). Moreover, glaucous bread wheat near isogenic lines produce significantly more wax and yield than its non-glaucous counterparts when exposed to drought (Guo et al., 2016). Additionally, Australian and American bread wheat varieties have also been seen to respond to drought with alkane accumulation. Under field conditions with reduced irrigation in Texas, winter wheat produced significantly more alkanes in the flag leaf (Liu et al., 2017). Similarly, Australian bread wheat varieties displayed significant increases in flag leaf alkane content when drought was applied in glasshouse conditions (Bi et al., 2017). Notably, most studies have focused on the flag leaf, which is the last leaf to develop and is responsible for producing 45%–58% of the photosynthates required for the grain-filling stage (Liu et al., 2018). These studies indicate that both β-diketone and alkane accumulation can improve bread wheat drought tolerance.

Previous studies have been performed on bread wheat varieties outside Canada (Guo et al., 2016; Bi et al., 2017; Liu et al., 2017). Yet, little is known about the wax responses of Canadian bread wheat varieties, despite their large contribution to the global wheat market. To leverage this knowledge, we focused on a CWRS panel, comprised of 30 wheat varieties based on historic popularity among growers, and released from 1842 to 2018. The varieties differed on their year of release, as well as on the ecozone they were bred for: Eastern (Manitoba), Western (Saskatchewan), or Founder (not bred for a specific region). We initially surveyed the cuticular wax composition of the 30 CWRS wheat varieties under field conditions. Following this, the two oldest and two most modern lines from each ecozone were subjected to drought in a greenhouse setting. Gene expression was then analyzed in four modern varieties under control conditions. We hypothesized that Western lines would outperform Eastern lines under drought conditions, as they were bred for a more arid climate. Additionally, we predicted that older varieties might outperform modern lines under drought, as beneficial traits for drought tolerance may have been lost in the pursuit of breeding for higher yield. Our results show that cuticular wax composition has remained stable; however, significant differences were detected in the accumulation of β-diketones. In contrast to older varieties, most modern varieties were unable to accumulate high levels of β-diketones under drought conditions suggesting that breeding for higher β-diketone accumulation in newer varieties could improve drought tolerance.

## 2 Materials and methods

### 2.1 Field experiment

Thirty CWRS wheat varieties were grown in the field at the Llewellyn Farm, Agriculture and Agri-Food Canada (AAFC), Saskatoon, Saskatchewan, from May to August 2021 (**Supplementary Table 1**) with five replicate plots per variety (n = 150 plots). To lessen the impact of seed age on germination, seeds from all 30 varieties were collected from field-grown plants the year before. The plots were seeded on 21 May 2021 at 263 seeds/m^2^, with five 4.26-m-long rows spaced 0.18 m apart, for a total seeded area of 3.79 m^2^. After plot trimming, the harvested area was 2.98 m^2^. Each plot was separated along its length by a row of fall rye, with buffer plots along the field’s border to account for border effects. The plots were rainfed, with 139.5 mm of rain falling during the growing season (Government of Canada, 2023). On 8 July, two fully expanded flag leaves were collected from the middle of each plot in 15-ml falcon tubes and placed in a dry ice container. The tubes were then shipped on dry ice to the University of Toronto Scarborough for cuticular wax analysis. Following the end of the growing season in August, grain was harvested and yield was expressed as the total weight of grain for each variety from each plot. The experiment was repeated the following year (2022) to collect plot yields. The presence of drought at the field site in 2021 and 2022 was tracked using the Canadian Drought Monitor, which provides a consolidated drought rating based on a five-category system (**Supplementary Figure 1**) (Government of Canada, 2023).

### 2.2 Greenhouse experiments

The two oldest and two most modern varieties from each ecozone were selected for a greenhouse drought experiment at the University of Toronto Scarborough (**Supplementary Table 1**). A 1:1 mixture of Promix HPCC Mycorrhizae and Promix PGX soil (PlantProducts, Ancaster, Ontario, Canada) was used to fill 3.8-L pots. Two seeds were planted in each pot, and four pots were grown for each variety. Plants were grown on a 14-h day cycle, with natural light supplemented with high-pressure sodium lights when natural light fell below 30,000 lx from 8:00 a.m. to 10:00 p.m. The drought treatment started 30 days after germination. Half of the pots were assigned to the drought treatment and received half the volume of water than plants in the control treatment. The quantity of water was adjusted as the plants grew to maintain a relatively constant water content in the soil throughout the experiment (**Supplementary Figure 2A**). Soil relative water content (RWC) was monitored twice a week for two random pots from each experimental condition using the gravimetric method (**Supplementary Figure 2B**) (Willick et al., 2018). Plant performance was measured in terms of tiller count, days to heading, final plant height, number of heads per plant, and head length at the end of each experiment. After 4 weeks of drought treatment, when plants were at the flowering stage, one flag leaf was collected from each pot for cuticular wax analysis. One representative plant from each experimental condition was photographed prior to flag leaf collection.

### 2.3 Cuticular wax extraction and analysis

Cuticular waxes were collected by submerging flag leaves in chloroform for 45 s. Tetracosane was added as the internal standard, with the quantity varying from 30 to 50 μg depending on the experiment. Following extraction, the wax extract was derivatized using N,O-bis(trimethylsilyl)trifluoroacetamide (BSTFA) and pyridine at 75°C for 60 min. Wax samples from the greenhouse drought experiment were analyzed using an Agilent 5977A Series GC/MSD System fitted with a 30-m × 0.25-mm × 0.25-μm HP-5 column. Samples were initially injected at 200°C and held for 1 min. The temperature was then increased to 320°C at a rate of 3°C/min and held at 320°C for 10 min. For all other samples, this method was translated to an Agilent 6890N GC/FID System fitted with a 30-m × 0.32-mm × 1-μm HP-1 column. For GC-MS analysis, peak areas were normalized to the specific amount of internal standard added to the sample and quantified using an external standard curve of pentadecanol for alcohols and an alkane mixture standard curve for all other compound classes. For GC-FID analyses, the internal standard was used for peak area normalization. Flag leaves were photographed following all wax extractions, and individual leaf area was determined using ImageJ (Wang et al., 2015b; Schneider et al., 2012). Compounds were grouped by compound class for statistical analysis. The total wax content was defined as the combined total of all compound classes, including all unidentified peaks.

### 2.4 Transcriptome analysis

To determine differences in gene expression across varieties, RNA was extracted from the flag leaf of AAC Tradition, AAC Concord, AAC Magnet, and AAC Starbuck plants grown under control conditions. Flag leaf samples were collected from three replicates from each variety for a total of 12 samples and immediately frozen in liquid nitrogen. Samples were shipped on dry ice to AAFC-Saskatoon, in Saskatchewan, Canada. RNA was extracted from 0.1 g of each sample using a Qiagen RNeasy Mini Kit and checked for quality using a Thermo Scientific NanoDrop™ One/OneC Microvolume UV-Vis Spectrophotometer before making libraries with the TruSeq UD V1.5 Kit. Samples were loaded on the Illumina NovaSeq 6000 platform for pair-end sequencing with 150 cycles at the Omics and Precision Agriculture Laboratory, Saskatoon, Canada. Reads were pre-processed by trimming the adaptor sequences, filtering low-quality reads, and eliminating short reads using Trimmomatic, with the argument ILLUMINACLIP: TruSeq3-SE:2:30:10 SLIDINGWINDOW:5:20 MINLEN:75 (Bolger et al., 2014). Due to the complexity of wheat genomes with multiple close homologs and homoeologs, Salmon, a quasi-mapping tool, was used to align the cleaned reads to the IWGSC RefSeq v2.1 complete reference genome database (Patro et al., 2017; Zhu et al., 2021). To find differences between varieties, a principal component analysis (PCA) was performed by the plotPCA function from DESeq2 (Love et al., 2014). To identify differentially expressed genes (DEGs), DESeq2 was used to perform pairwise comparisons between varieties (DEGs defined as log2 fold change >1 or <−1, adjusted p-value < 0.05). Next, to find expression markers, a targeted gene approach that focused on genes previously implicated in the three wax biosynthetic pathways in wheat was taken (**Supplementary Table 2**).

### 2.5 Statistical analysis

For the cuticular wax analysis, normality was assumed according to the central limit theorem for sample sizes larger than 30 (Kwak & Kim, 2017). The equal variance assumption was met if it passed either the Bartlett or Levene tests for homogeneity of variances; in the cases where it did not, data were log transformed and re-tested. The field experiment was analyzed as a two-way ANOVA with ecozone and year as categorical independent variables, with varieties released after 2000 being considered modern. Similarly, the greenhouse experiment was analyzed as a three-way ANOVA adding drought treatment as the third variable. The morphological data were analyzed following the same procedure, except for plant height that required non-parametric Kruskal–Wallis tests. For the expression analysis, a one-way ANOVA, alongside a Tukey HSD test, was applied on the transcripts per million (TPM) values of the wax genes and wax component quantities to determine differences based on variety. Statistical analysis was performed on MVApp and R-studio using the rstatix and dplyr packages (Julkowska et al., 2019; Kassambara, 2023; RStudio Team, 2020; Wickham et al., 2023).

## 3 Results

### 3.1 Yield in a historical collection of wheat germplasm is impacted by environmental conditions

**Figure 1:**
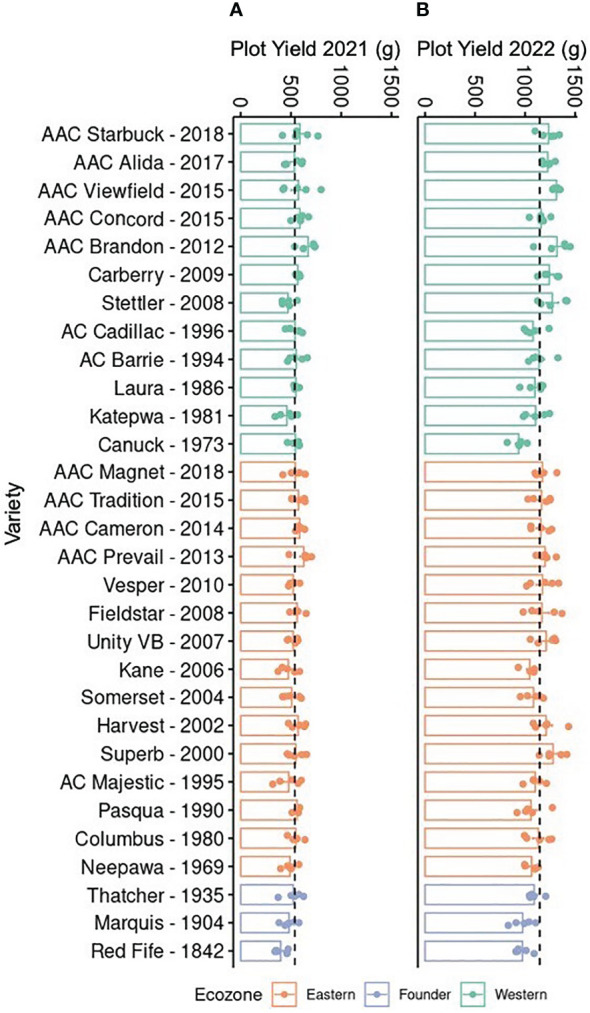
Yield of 30 Canadian Western Red Spring wheat varieties. Plants were grown in Saskatoon, Saskatchewan, from May to August in **(A)** 2021 and **(B)** 2022. Bars show the mean and standard error (n = 5, shown as individual points); dotted lines indicate the mean across all samples. Varieties are arranged in reverse chronological order by ecozone. The yield was measured in grams for each plot.

Drought events have been shown to coincide with reduction in wheat yield; hence, it is important to identify germplasm that maintains relatively stable yields despite challenging environmental conditions (McCaig and DePauw, 1995). As a first step toward this goal, the yield of a historical collection of CWRS wheat varieties was compared over 2 years. These varieties had been bred for two different Canadian ecozones, with the Eastern ecozone being more moist than the Western (Grant et al., 2012; He et al., 2018). Although varieties were selected at two sites, Western and Eastern varieties have been developed by repeated crossing with a few common lines like Neepawa, Thatcher, and Columbus (**Supplementary Table 1**). Hence, they might still share some pedigree even when bred for different conditions. Nevertheless, we anticipated better yields for the modern varieties given the gains obtained through breeding, particularly for the local Western varieties when grown at Saskatchewan. Comparisons over two growth seasons showed a twofold difference in yield, with varieties in 2021 producing 538.77 ± 7.21 g, compared to 1145.85 ± 10.83 g in yield in 2022 (**Figure 1**). Interestingly, the lower yield in 2021 coincided with drier conditions at the field site implying the impact of drought stress on wheat yield (**Supplementary Figure 1**) (Government of Canada, 2023). Furthermore, varieties from both ecozones were negatively affected (**Figure 1A**). Yet under near optimal growth conditions in 2022, the increase in yield in more modern varieties was evident for the local Western varieties (**Figure 1B**). These observations suggest that, although breeding has been successful in improving productivity, crop resilience is not necessarily present in modern varieties.

### 3.2 Variation in cuticular waxes seen in quantity not quality

**Figure 2:**
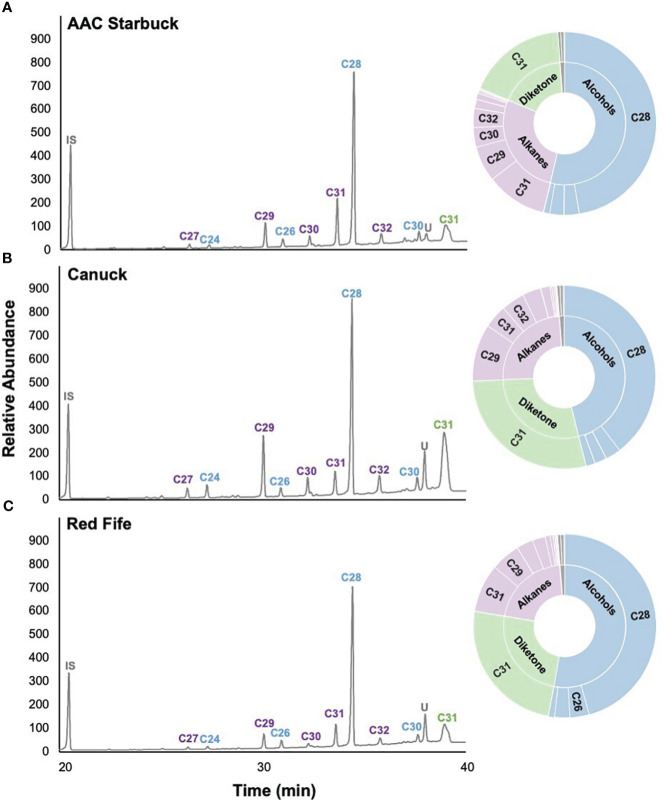
Representative cuticular wax profiles of flag leaves of wheat varieties from different years of release. GC-FID chromatograms of **(A)** AAC Starbuck, **(B)** Canuck, and **(C)** Red Fife. The percentage composition is shown on the sunburst plot. Each compound class is identified with a different color (blue: primary alcohols, purple: alkanes, green: β-diketones, gray: unknown) with the number corresponding to the number of carbons in the molecule. The most predominant compounds are labeled on the chromatograms with the color of the label corresponding to the compound class. IS, internal standard; U, unknown.

Since cuticular wax composition has not actively been selected for breeding programs in Canada, we hypothesized that chemical diversity in the wax components might have been lost over the years. To investigate this, we profiled the cuticular waxes of flag leaves grown during the 2021 season. Several wax compound classes corresponding to the three biosynthetic pathways in wheat were identified: odd-chain alkanes (C23–C33), primary alcohols (C24–C30), fatty acids (C14–C26), and a C31 β-diketone (**Figure 2**). The predominant compound class for most varieties was primary alcohols (accounting for 25.1%–78.3% of the total wax load), particularly octacosanol (19.7%–64.0% contribution), followed by odd-chain alkanes (6.2%–39.0% contribution), β-diketones (2.1%–41.8% contribution), and fatty acids (0.4%–2.2% contribution). When comparing all 30 varieties, the compounds identified were the same, with some differences in the contribution of each class, as seen when comparing AAC Starbuck (the most modern Western variety), Canuck (the oldest Western variety), and Red Fife (Canada’s oldest founder variety) (**Figures 2A–C**). Moreover, no significant differences were observed for any of the compound classes between ecozones (**Table 1**; **Supplementary Figure 3**). Additionally, even the Founder varieties show similar variation and composition to the Canadian bred varieties. However, the quantity of each wax compound class differed significantly when varieties were compared based on the year of release (**Table 1**). This indicates that, although there is limited chemical diversity in wax composition in the CWRS collection, the quantity and contribution of each compound class has changed over the years.

**Table 1:** Statistical analysis for the wax analyses from the field.

Variable	DF	β-diketone	Odd-chained alkanes	Primary alcohols	Fatty acids	Total wax
Ecozone	1	4.013 (0.047)	0.065 (0.799)	0.641 (0.425)	0.400 (0.528)	0.064 (0.801)
Year of release	1	18.220 (3.76e−05**)	7.233 (0.008*)	0.477 (0.491)	8.488 (0.004*)	8.640 (0.004*)
Ecozone × year of release	1	0.266 (0.607)	0.528 (0.469)	0.001 (0.980)	0.169 (0.682)	0.417 (0.520)

Degrees of freedom (DF), F-values, and p-values (in brackets) are shown for a two-way ANOVA using ecozone and year of release for the field experiment. Significance (*p-value < 0.01, **p-value < 0.001) is indicated next to each significant p-value.

**Figure 3:**
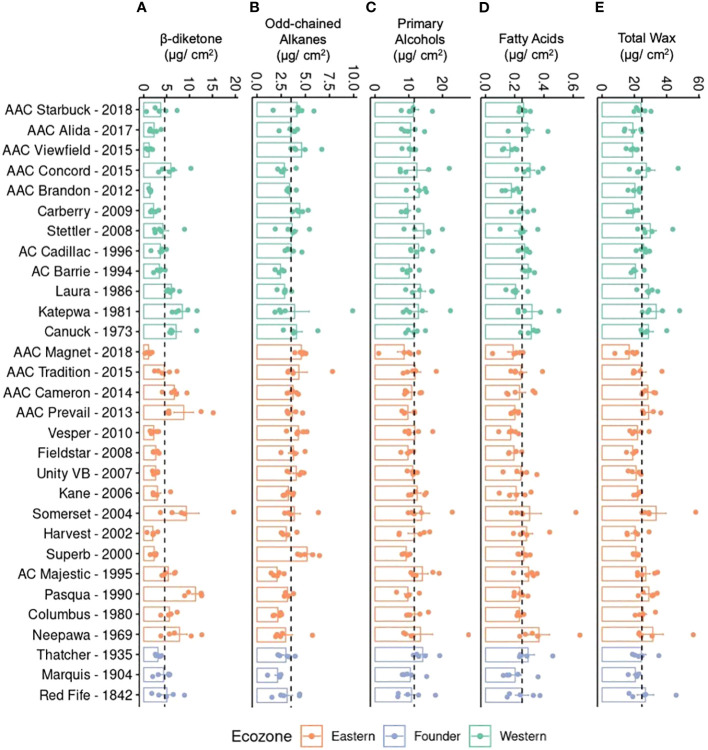
Cuticular wax composition of flag leaves in a field setting. Bar plots showing averages and standard errors (n = 5, shown as individual points) for **(A)** β-diketones, **(B)** odd-chained alkanes, **(C)** primary alcohols, **(D)** fatty acids, and **(E)** total wax for 30 CWRS wheat varieties grown in 2021 in Saskatoon, Saskatchewan. Dotted lines indicate the mean across all samples for that particular trait. Waxes were extracted from flag leaves using chloroform and quantified using gas chromatography and flame ionization detection. Varieties are arranged in reverse chronological order by ecozone (light green: Western, orange: Eastern, periwinkle: Founder).

To investigate if the differences in quantity are due to one or all compounds changing in accumulation, the total quantity of each compound class was plotted (**Figure 3**). Unlike primary alcohols, a large variation was found in the accumulation of β-diketones, with older varieties producing, on average, significantly higher levels (**Figure 3A**). Older varieties also accumulated more wax, on average, than modern varieties indicating that modern varieties have reduced β-diketones and total wax compared to older germplasm (**Figure 3E**). This overall trend was observed in varieties from both ecozones. Overall, a twofold difference was seen between the variety accumulating the most wax per surface area (Somerset, Eastern variety released in 2004) and the least wax (AAC Magnet, Eastern variety released in 2018) (**Figure 3E**). While modern varieties tend to have lower levels of β-diketones, they have significantly higher levels of odd-chained alkanes (**Figure 3B**). Modern lines, such as AAC Viewfield from the Western ecozone and AAC Magnet from the Eastern ecozone, had a notably higher alkane content compared to older lines. To test if any of the wax classes has an effect on yield, a correlation analysis was performed between yield and each compound class, but no clear associations were found. It is worth noting that during the 2021 field season, no major differences were observed in yield in the historic collection. Therefore, the lack of association might be a result of the reduced variability in yield in 2021. Altogether, these results indicate that during the breeding history of CWRS wheat, cuticular wax composition has remained fairly constant, except for significant differences in the accumulation of two compound classes. Older varieties accumulated more β-diketones and wax overall, whereas modern varieties accumulated more odd-chained alkanes.

### 3.3 Drought treatment impacts wheat growth and cuticular wax load

The field observations indicated that the historic collection has variation in the quantity of β-diketones, odd-chained alkanes, and total wax load. Additionally, the 2021 growing season was drier, which negatively affected the yield of all varieties. This prompted the question on how CWRS wheat varieties respond to drought stress. To address this, a drought experiment was performed with eight varieties representing the two oldest and two most modern varieties at each ecozone: Neepawa, Columbus, AAC Tradition, AAC Magnet from the Eastern ecozone; and Katepwa, Canuck, AAC Starbuck, and AAC Concord from the Western ecozone. Plants were allowed to grow under control conditions for 30 days, after which half of the pots received 50% of the water given to controls. Soil RWC measurements confirmed that plants assigned to the drought treatment had lower water content (**Supplementary Figure 2B**). After a month under these conditions, the negative effects of drought were seen across several traits. Drought-treated plants had visibly less aboveground biomass than control-treated plants (**Supplementary Figure 4**), and they formed significantly fewer tillers and heads than well-watered plants (**Supplementary Figures 5A–E**), which will affect their yield. Yet, the water regime used here did not significantly affect time to flowering, plant height, or head length. Even though Western varieties have been bred to withstand a more arid climate than Eastern varieties, similar responses to drought were observed. The results indicate that in terms of growth, older varieties produced a higher number of heads but shorter in length than modern varieties (**Supplementary Figures 5D, E**). It is worth noting that modern breeding efforts have predominantly focused on obtaining higher yield and pathogen resistance; hence, we expected modern varieties would be more sensitive to drought.

**Figure 4:**
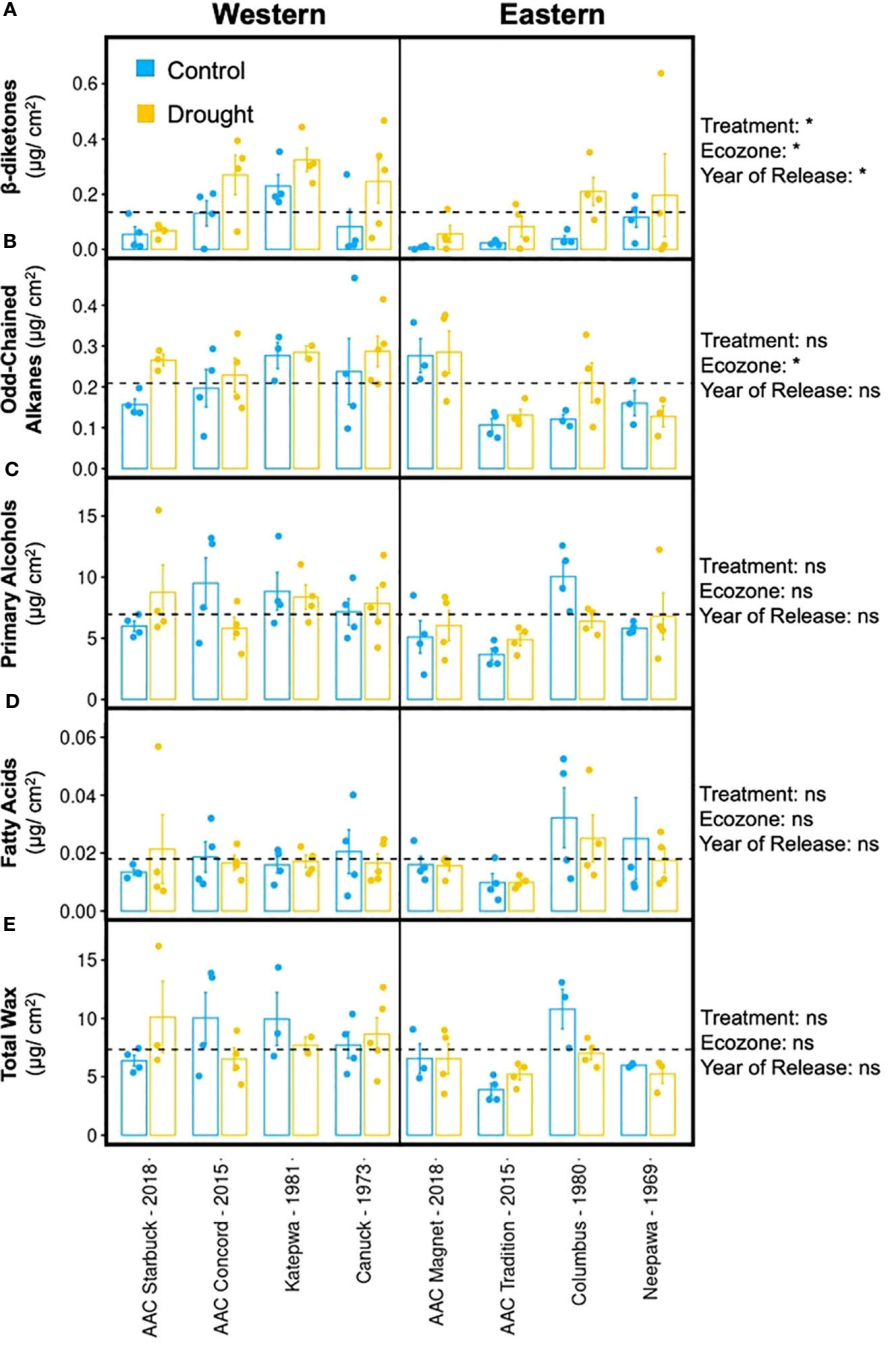
Flag leaf cuticular wax composition upon drought treatment. Averages and standard error (n = 4, shown as individual points) and results of a multivariate regression using treatment, ecozone, and year of release (modern versus old) as variables (ns, p-value > 0.01, *p-value < 0.01) are shown for a **(A)** β-diketone, **(B)** odd-chained alkanes, **(C)** primary alcohols, **(D)** fatty acids, and **(E)** total wax for eight CWRS varieties grown in a greenhouse. Waxes were extracted from flag leaves using chloroform and quantified using gas chromatography and mass spectrometry. Light blue: control conditions, yellow: drought treated. Dotted lines indicate the mean across all samples for that particular trait.

The formation of tillers and heads was negatively affected by drought in most varieties, apart from AAC Concord and Neepawa. Hence, similar responses were also expected in terms of their cuticular waxes (**Figures 4A–E**). Surprisingly, the wax response to drought was different across varieties. Whereas AAC Tradition and AAC Starbuck responded to the treatment by increasing their total wax load, AAC Concord, Katepwa, and Columbus showed the opposite response (**Figure 4E**; **Supplementary Figure 6E**). Despite the variable changes in wax load, a consistent increase in β-diketone accumulation with drought was observed across varieties, while the magnitude of the increase varied from 19.3% in AAC Starbuck to 74.9% in Columbus (**Figure 4A**; **Supplementary Figure 6A**). Even though the β-diketone content increased in drought-treated samples, visible differences in glaucousness were not observed on the abaxial surface of the flag leaf, except for Katepwa (**Supplementary Figure 7**). Interestingly, in the varieties that showed a reduction in total wax load with the treatment, the reduction in primary alcohols was responsible for the observed decrease in total wax (**Figure 4C**).

When comparing varieties from both ecozones, Western varieties had, on average, significantly higher β-diketone, alkane, and overall wax content compared to Eastern varieties (**Supplementary Figure 8A**). This aligns with the hypothesis that Western varieties are better suited to withstand drought conditions given that they have been selected in a more arid climate. Older varieties also accumulated significantly more β-diketones than modern varieties suggesting that older varieties might possess higher stress tolerance relative to newer varieties (**Supplementary Figure 9A**). AAC Magnet showed minimal differences in wax content between control and drought-treated conditions for all compound classes. Unlike AAC Concord and AAC Magnet, both AAC Tradition and AAC Starbuck shared similar wax responses to drought and produced higher wax content for all compound classes. The results indicate that, in terms of cuticular wax composition and load, older varieties show higher induction of β-diketone accumulation upon drought conditions, a trait that will become important under future climate scenarios.

### 3.4 Gene expression analysis supports differences between modern varieties

**Figure 5:**
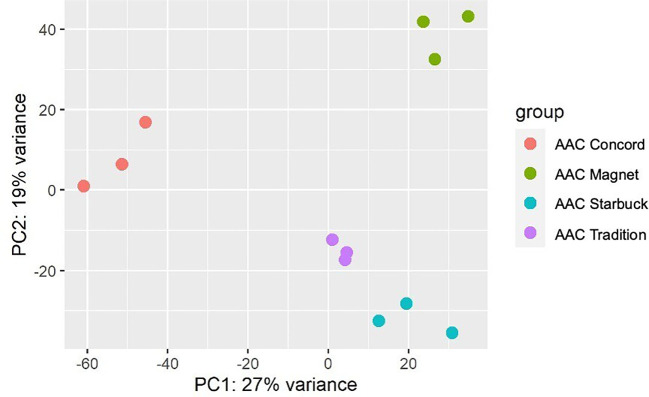
Principal component analysis of gene expression data. Three flag leaf samples from four modern CWRS wheat varieties were used for a transcriptome analysis. Plants were grown under control conditions in a greenhouse setting and flag leaves collected during the flowering stage.

**Figure 6:**
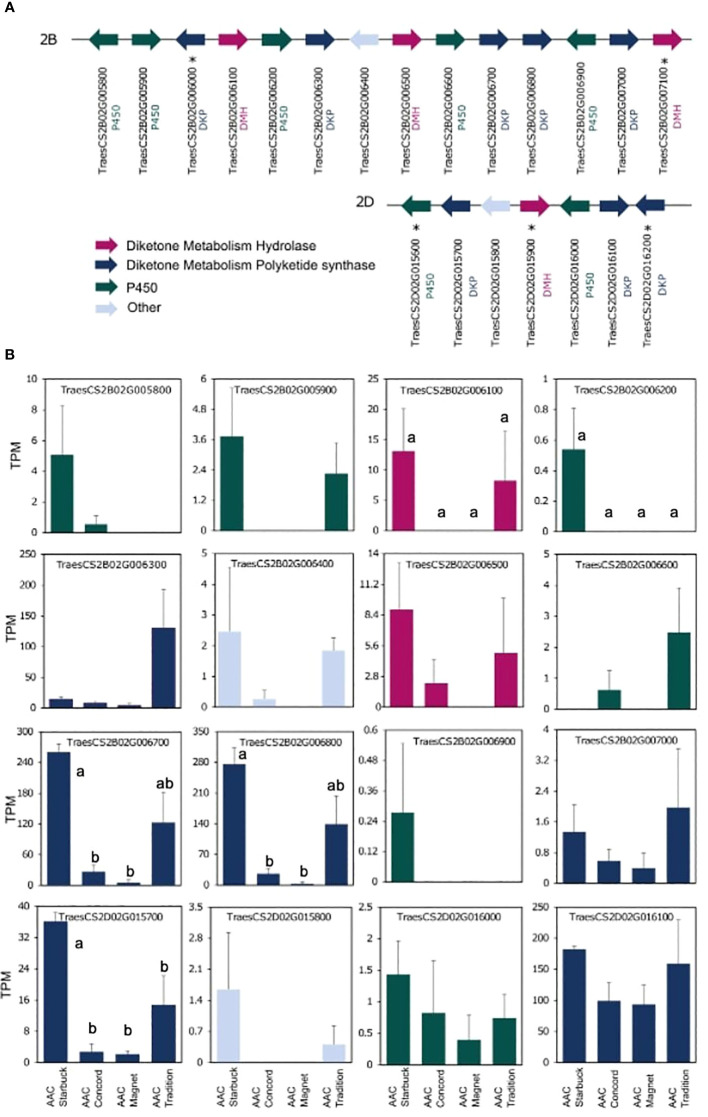
Gene expression of the *W1* locus controlling β-diketone accumulation. Sequences from the previously characterized *W1* locus from the cultivar Zavitan were used to conduct BLASTP searches in the IWGSC assembly (Hen-Avivi et al., 2016). **(A)** Structure of the two clusters identified on chromosomes 2B and 2D. Genes and distances between genes are not drawn to scale. Genes not expressed are indicated with an *. **(B)** Expression levels plotted as TPM for each of the four varieties. A one-way ANOVA was performed to test for differences in expression across varieties (**Supplementary Table 3**). When significant, it was followed by a Tukey’s HSD test. Letters above the bars indicate which pairwise comparisons are significantly different (p-value < 0.05).

**Figure 7:**
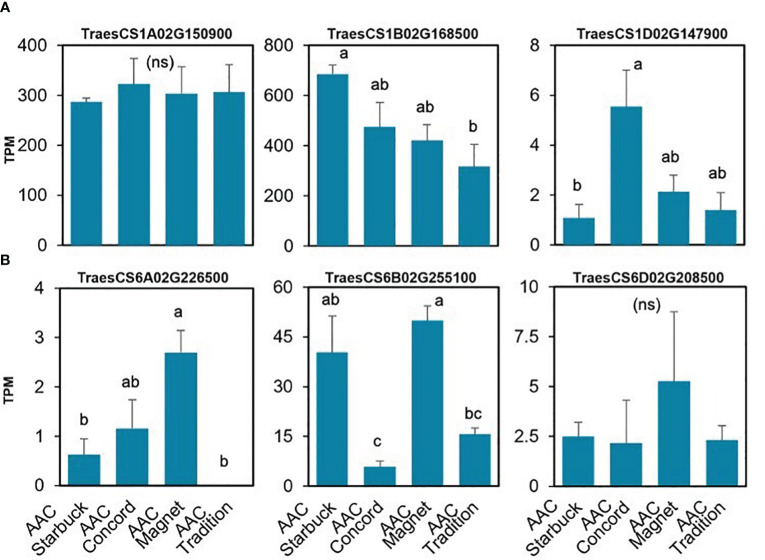
Gene expression of the *TaCER1* genes involved in alkane accumulation. **(A)** *TaCER1–1* homologs (Li et al., 2019). **(B)** *TaCER1–6* homologs (He et al., 2022). Average expression and standard error for the three replicates per variety are plotted in transcripts per million (TPM) units. A one-way ANOVA was performed to test for differences in expression (**Supplementary Table 3**). When significant, it was followed by a Tukey’s HSD test. Letters above the bars indicate which pairwise comparisons are significantly different (p-value < 0.05; ns, not significant).

The data presented above show that, although wheat varieties have similar cuticular wax composition, they respond differently to drought stress. To assess if the differences are also seen at the gene expression level, the transcriptome of the flag leaf of four modern varieties grown under control conditions was investigated. A principal component analysis (PCA) segregated the samples by variety indicating that there are overall distinct patterns of gene expression (**Figure 5**). To incorporate cuticular wax traits in future breeding programs, it would be helpful to identify expression markers to accelerate the selection of individuals that have desirable wax traits. We used a targeted gene approach focusing on genes previously implicated in the three wax biosynthetic pathways in wheat (**Supplementary Table 3**). Using the reported sequences from the wheat accession Zavitan (Hen-Avivi et al., 2016) as queries for BLASTP searches on EnsemblPlants, we were able to identify several isoforms for the previously characterized genes (**Supplementary Table 3**). VLCFAs are reduced into primary alcohols by FAR. Across all varieties screened, C28 primary alcohol was the dominant wax component, with lower quantities of C24, C26, and C30 alcohols (**Figure 2**). Three homologs of *TaFAR4*, one from each sub genome, were found to be expressed in all cultivars. This was followed by lower expression levels for one *TaFAR2*, while one *TaFAR5* was exclusively expressed in AAC Magnet and AAC Tradition. The biosynthesis of β-diketones and related compounds is controlled by the *W1* locus. A cluster on chromosome 2B and a smaller on 2D were found in IWGSC RefSeq v1.1. with a total of eight *DKP*s, four *DMHs*, and seven P450s [The International Wheat Genome Sequencing Consortium (IWGSC) et al., 2018] (**Figure 6**). Large variation in expression levels was observed across *DKP*-encoding genes from low expression in *TraesCS2B02G007000* to high levels in *TraesCS2D02G016100*. Additionally, some genes were differentially expressed between accessions, like *TraesCS2B02G006800* (**Supplementary Table 3**). Interestingly, an overall pattern can be seen across the expression of the different genes, with higher expression in AAC Starbuck and AAC Tradition, the two varieties with slightly higher accumulation of C31 β-diketone (**Supplementary Figure 10A**). This could indicate that the genes in the cluster are not only physically linked but also their expression is coordinately regulated. This confirms the strong effect of the *W1* locus on the levels of β-diketones, which makes it a promising marker for breeding. AAC Magnet accumulated more alkanes than the other varieties, yet the expression of *TaCER1–1* genes did not follow the same pattern. Instead, *TaCER1–6* genes, particularly the gene from the A sub genome *TraesCS6A02G226500*, followed the pattern of accumulation of alkanes (**Figure 7**). Taken together, the analysis indicates that the expression of these genes could be used to select germplasm that accumulates a higher wax load. The next step would be to identify single-nucleotide polymorphisms that predict the expression of these genes for marker-assisted selection.

## 4 Discussion

The repetitive use of the same germplasm for crossing leads to genetic bottlenecks in domesticated material (Metakovsky et al., 2019). This could be seen in the homogenous composition of the flag leaf cuticular waxes of the 30 CWRS wheat varieties, even though Red Fife was introduced in approximately 1842. Although cuticular waxes were not intentionally selected for or against, the early observations that found glaucous appearance associated with drought resistance might have favored the indirect selection of varieties with more dense wax crystals and hence higher wax load. Nevertheless, this was not the case for CWRS wheat varieties, and instead, a lower total wax load and diketone composition was seen in modern varieties. It is worth noting that similar wax profiles have been previously reported for wheat germplasm elsewhere, such as varieties bred for Southern Australia (Bi et al., 2017) highlighting the narrow genetic diversity in cereal crops even in a trait that has not been under direct selection. Furthermore, varieties released for the Eastern and Western ecozones in Canada have undergone independent breeding efforts, yet no significant differences in cuticular wax, apart from odd-chained alkanes, were seen when all varieties were grown under the same field conditions (**Table 1**). Instead, differences could be observed in the quantity of the individual compound classes, particularly in the β-diketone, 14,16-hentriaconta-dione. The higher accumulation of β-diketones in older varieties was clear in the field and under greenhouse settings. In contrast, modern varieties have a higher alkane content.

To investigate the variation in β-diketones further, eight varieties were chosen for a greenhouse drought experiment. The common feature in all varieties was the increased accumulation of β-diketones upon reduced watering. Yet, the increase did not always result in a higher total wax load. This could be explained by the accompanying decrease in primary alcohols in AAC Concord, Katepwa, and Columbus upon drought stress. One possible explanation is that there could be limitations on the total carbon flux into cuticular waxes. To achieve higher levels of alkanes and β-diketones under drought conditions, it might be necessary to redirect the precursor VLCFAs used in the alcohol-forming pathway into the other two pathways. Hence, finding wheat germplasm with reduced primary alcohols, such as Pasqua (released in 1990 for the Eastern ecozone), might be worth investigating.

In addition to minimizing non-stomatal water loss, cuticular waxes can assist with reflecting harmful radiation, such as UV-B, UV-C, and near infrared (Mulroy, 1979; Balouchi et al., 2009), and reflecting light in the 330- to 680-nm range (Camarillo-Castillo et al., 2021). When surface waxes were removed on the abaxial and adaxial surface of wheat leaves, reflectance was reduced by twofold on the abaxial surface relative to the adaxial surface (Uddin and Marshall, 1988). However, it is important to note that, although all wax compounds are hydrophobic, the functional groups that decorate them are known to affect their physical and chemical properties, for example, alcohol-rich waxes form platelet-shaped crystals, whereas β-diketones form tubule-shaped crystals (Su et al., 2020). The morphology of the crystals can be associated with different attributes in light reflection. Moreover, fatty acids have been shown to create poor hydrophobic barriers in both natural and artificial membranes (Bi et al., 2017), whereas alkanes have been reported to minimize water diffusion through the cuticular membrane and increase mesophyll conductance (Kosma et al., 2009; Su et al., 2020). Hence, the properties of the cuticular wax layer can be adjusted by changing the contribution of individual compounds. Modern CWRS wheat varieties had a higher accumulation of alkanes. Similarly, Australian bread wheat and American winter wheat varieties have been observed to respond to drought stress via alkane accumulation (Bi et al., 2017; Liu et al., 2017). Another study that compared wax responses to drought in glaucous and non-glaucous Chinese bread wheat varieties found that non-glaucous varieties were more likely to respond to drought with higher alkane accumulation, while glaucous varieties responded with higher β-diketone levels (Su et al., 2020). Similarly, in this study, AAC Starbuck did not increase the accumulation of β-diketones with drought, but higher levels of alkanes, alcohols, and fatty acids were observed. This suggests that the glaucous and non-glaucous responses reported in Chinese varieties are conserved in Canadian varieties. Most varieties responded by increasing the accumulation of β-diketones, but when this was not possible, other compound classes, including alkanes, can be upregulated. Therefore, breeding for higher wax content might be complicated by competition between the three wax biosynthetic pathways in wheat and the particular properties conferred by each functional class.

Understanding the functional role of each compound class under stress conditions could assist in breeding a better cuticular wax for future climates. The wheat ideotype would have the capacity to induce a large increase in β-diketone accumulation upon drought treatment, while maintaining a stable yield. The greenhouse experiment allowed us to identify material that meets this criterion. AAC Concord was notable in this respect showing a large increase in β-diketone accumulation and no negative effects in growth with reduced water. Although there are other varieties with higher yield, the tolerance of AAC Concord to reduced water is a desirable trait that could be incorporated in breeding programs. This is in agreement with prior studies that have observed that glaucous wheat varieties containing high levels of β-diketones outperform non-glaucous varieties under drought (Guo et al., 2016; Hunt et al., 2018). Some of the effects of higher β-diketone content are not a mere consequence of increasing the cuticular wax load. For example, the glaucous Chinese bread wheat variety HY 2912 increased β-diketone deposition, which, in turn, allowed the plants to maintain high stomatal and mesophyll conductance and higher intercellular CO_2_ concentration. It has been observed that the tubule structure of β-diketone crystals reduces CO_2_ diffusion from the mesophyll to the atmosphere (Su et al., 2020). This implies that drought tolerance is linked to β-diketone deposition. Based on this, reincorporating a higher and inducible β-diketone content into modern varieties that have a low β-diketone content, such as AAC Magnet, may improve drought responses (**Figure 3A**). The RNASeq results suggest that AAC Magnet exhibits low expression for DMPs, which may explain why less β-diketones were observed in AAC Magnet flag leaf samples (**Supplementary Table 3**). Increasing DMP expression in AAC Magnet may, in turn, increase β-diketone accumulation in this variety. However, it is also important to note that AAC Brandon, which showed the highest yield in 2021 and 2022, accumulates very low quantities of β-diketones. Clearly, other mechanisms that provide tolerance to stress are at play, and cuticular waxes should be incorporated in breeding programs as an additional trait, not in replacement of others.

In terms of understanding how the cuticular wax responses are orchestrated at the gene level, future experiments should look at early and late time points upon application of the stress to check the induction/repression of wax biosynthetic genes. To move forward with the screening of waxes in new germplasm, having genetic markers that aid in the identification of elite material will be crucial. Three main wax biosynthetic pathways are present in wheat and other cereal crops, with key enzymes previously characterized (Wang et al., 2015b; Hen-Avivi et al., 2016; Wang et al., 2016; Li et al., 2019; He et al., 2022). Primary alcohols were the dominant compound class in flag leaves, and several *FAR* genes were found expressed in CWRS germplasm. Unexpectedly, *TaFAR3*, which produces C28 primary alcohols when expressed in yeast, was not expressed in any of the four modern varieties studied here (Wang et al., 2016). In contrast, homologs of *TaFAR4* from each sub genome were highly expressed in the flag leaves analyzed, even when the *in vitro* activity of *TaFAR4* is to synthesize C24 alcohols (**Supplementary Table 2**). This highlights the need to test the carbon length preference of additional *TaFAR* alleles to determine how fast substrate specificity can change.

Results from the expression analysis of flag leaves presented here and in past work suggest that the *W1* locus could be an effective marker for selecting varieties with high β-diketone content (Hen-Avivi et al., 2016). The fact that the three enzymes required for β-diketone synthesis (DMH, DKP and P450s) are physically linked in the same locus facilitates their selection. Moreover, the high β-diketone content in AAC Tradition and AAC Starbuck was closely matched by the expression levels of the genes within the *W1* locus indicating that not only are the genes physically linked, but their expression is too. Particularly, the higher expression levels of PKS-encoding genes suggest that they encode the rate-limiting step in the biosynthesis of β-diketones, whereas P450-encoding genes had the lowest levels of expression (**Figure 6**). Interestingly, recent studies have found that AAC Tradition differs in the position of the hydroxyl group that decorates β-diketones in the spike cuticular waxes (Chen et al., 2023). The P450s within the *W1* locus are good candidates for explaining this chemical phenotype.

## 5 Conclusion

Due to accelerated climate change, there is an urgency for drought-tolerant bread wheat varieties to meet global food demands. Evidence from this study in Canadian germplasm and work from other groups suggest that increasing β-diketone accumulation can assist with increasing drought tolerance in wheat. However, both field and greenhouse experiments indicate that this response is reduced in modern lines. Through selective breeding for a stronger β-diketone response in the flag leaves of future CWRS varieties, wheat breeders may be able to develop more resilient varieties that are capable of withstanding future climate scenarios.

## Data Availability

The RNAseq datasets generated for this study can be found in the GEO repository under accession number GSE254797. The R-code used in this study can be found in https://github.com/wheat0624/Frontiers-in-Science-Wheat-Paper-Code-Repository.git.
