# Supplementary material for: β-diketone accumulation in response to drought stress is weakened in modern bread wheat varieties (Triticum aestivum L.)
Source: Front Plant Sci. 2024 Aug 9;15:1401135. doi: 10.3389/fpls.2024.1401135 (PMC11341480; doi:10.3389/fpls.2024.1401135)
Supplement: Supplementary file 1 [file Presentation_1.pptx]

## Slide 1
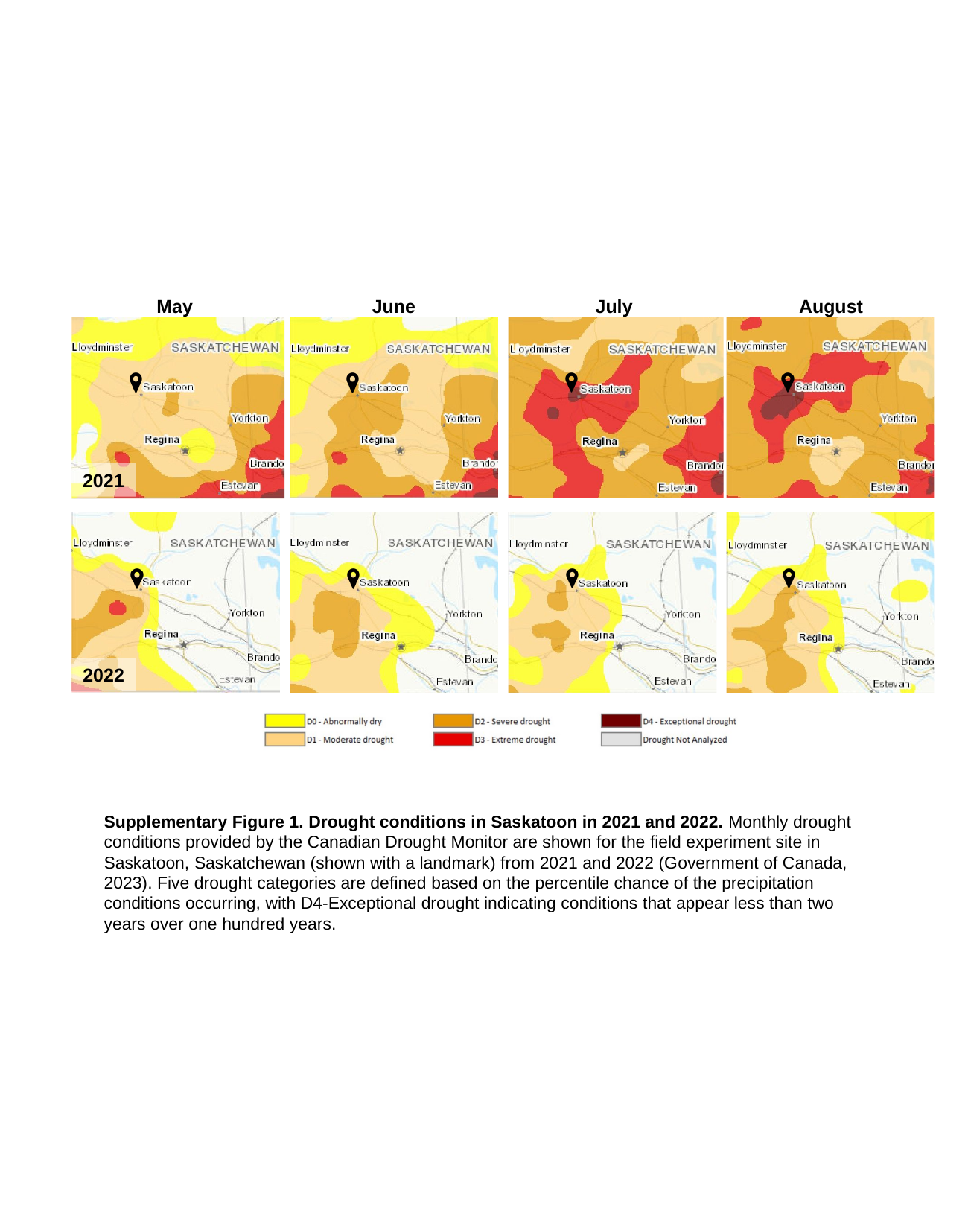

May
June
July
August
2021
2022
Supplementary Figure 1. Drought conditions in Saskatoon in 2021 and 2022. Monthly drought conditions provided by the Canadian Drought Monitor are shown for the field experiment site in Saskatoon, Saskatchewan (shown with a landmark) from 2021 and 2022 (Government of Canada, 2023). Five drought categories are defined based on the percentile chance of the precipitation conditions occurring, with D4-Exceptional drought indicating conditions that appear less than two years over one hundred years.

## Slide 2
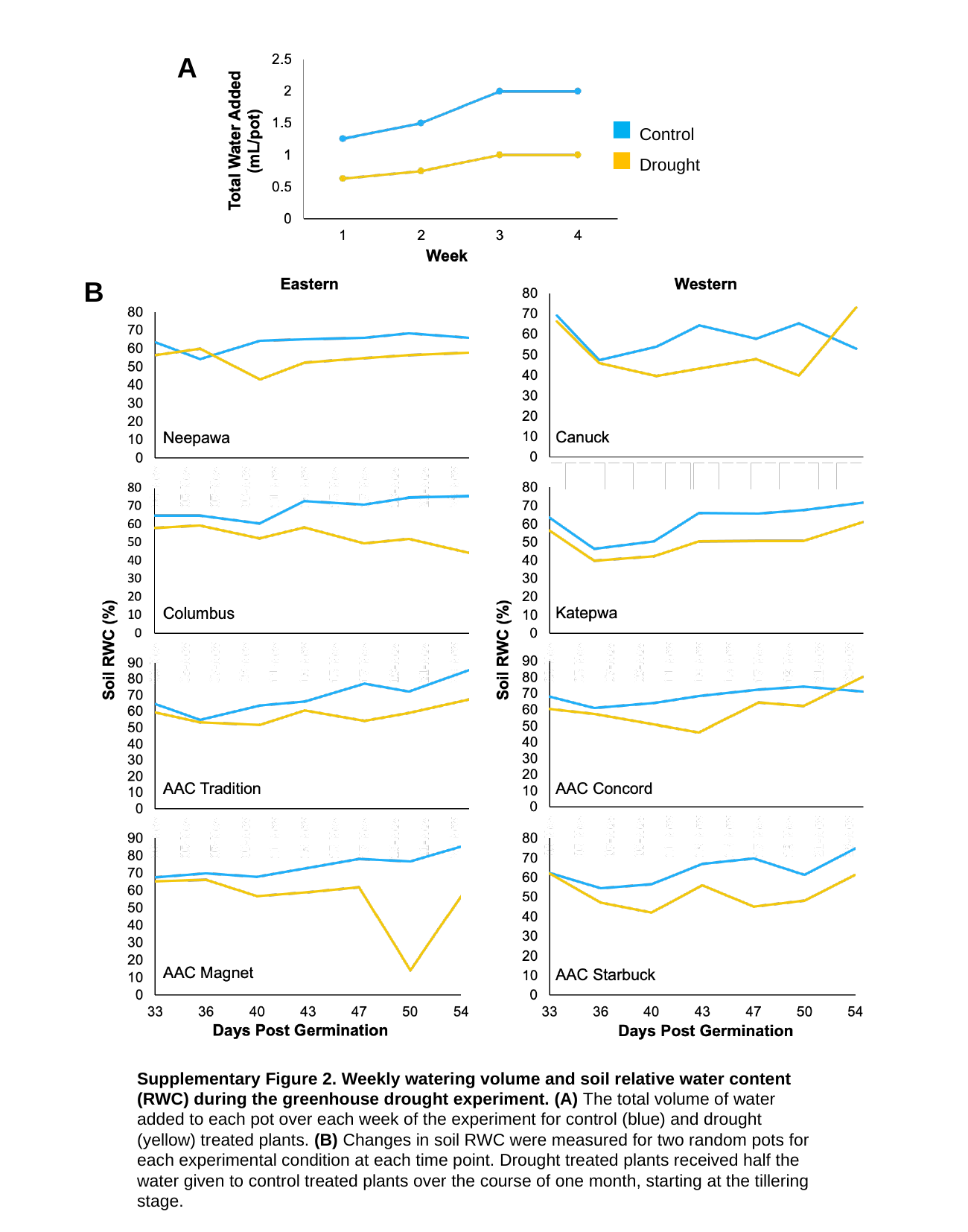

A
Control
Drought
B
Supplementary Figure 2. Weekly watering volume and soil relative water content (RWC) during the greenhouse drought experiment. (A) The total volume of water added to each pot over each week of the experiment for control (blue) and drought (yellow) treated plants. (B) Changes in soil RWC were measured for two random pots for each experimental condition at each time point. Drought treated plants received half the water given to control treated plants over the course of one month, starting at the tillering stage.

## Slide 3
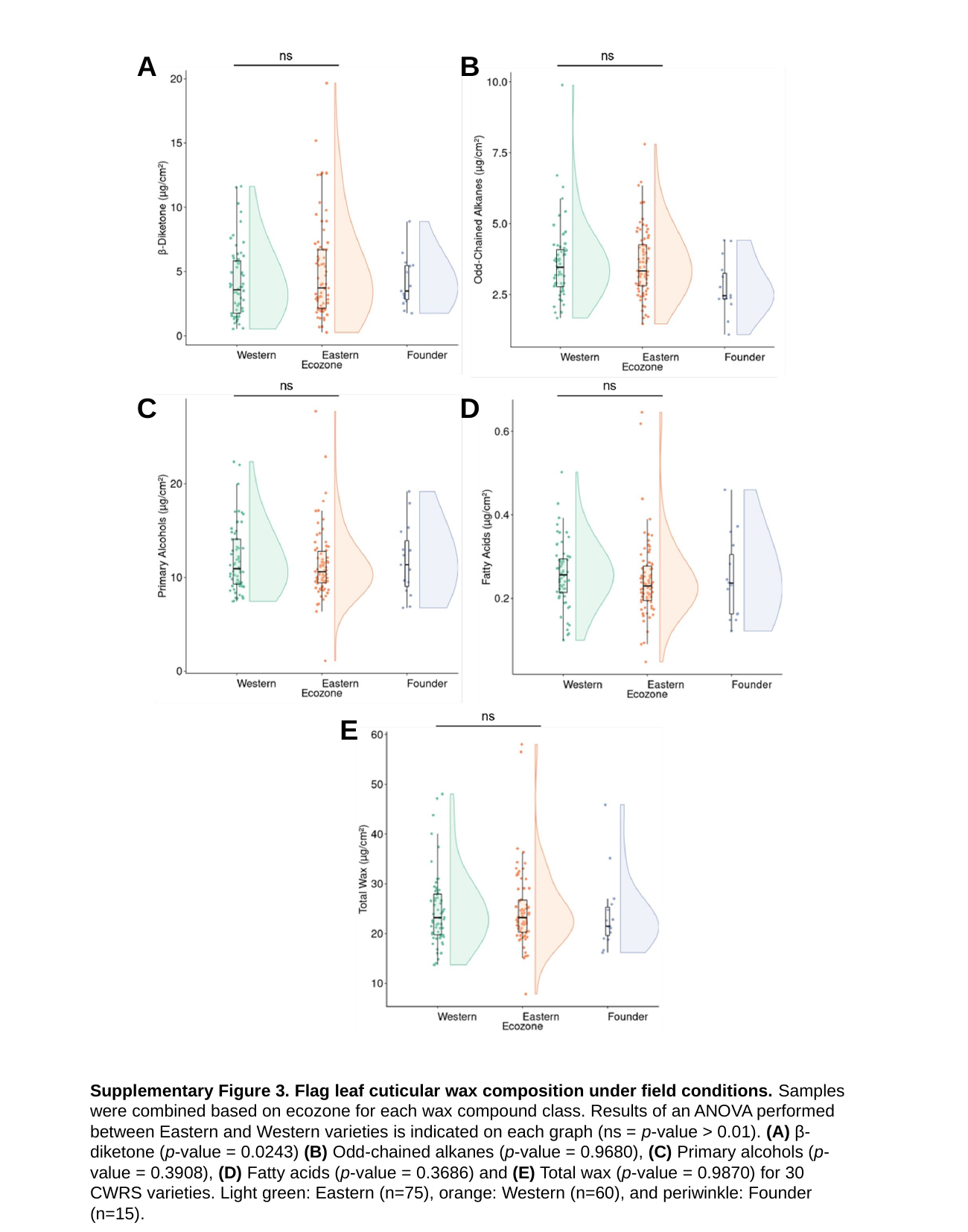

A
B
C
D
E
Supplementary Figure 3. Flag leaf cuticular wax composition under field conditions. Samples were combined based on ecozone for each wax compound class. Results of an ANOVA performed between Eastern and Western varieties is indicated on each graph (ns = p-value > 0.01). (A) β-diketone (p-value = 0.0243) (B) Odd-chained alkanes (p-value = 0.9680), (C) Primary alcohols (p-value = 0.3908), (D) Fatty acids (p-value = 0.3686) and (E) Total wax (p-value = 0.9870) for 30 CWRS varieties. Light green: Eastern (n=75), orange: Western (n=60), and periwinkle: Founder (n=15).

## Slide 4
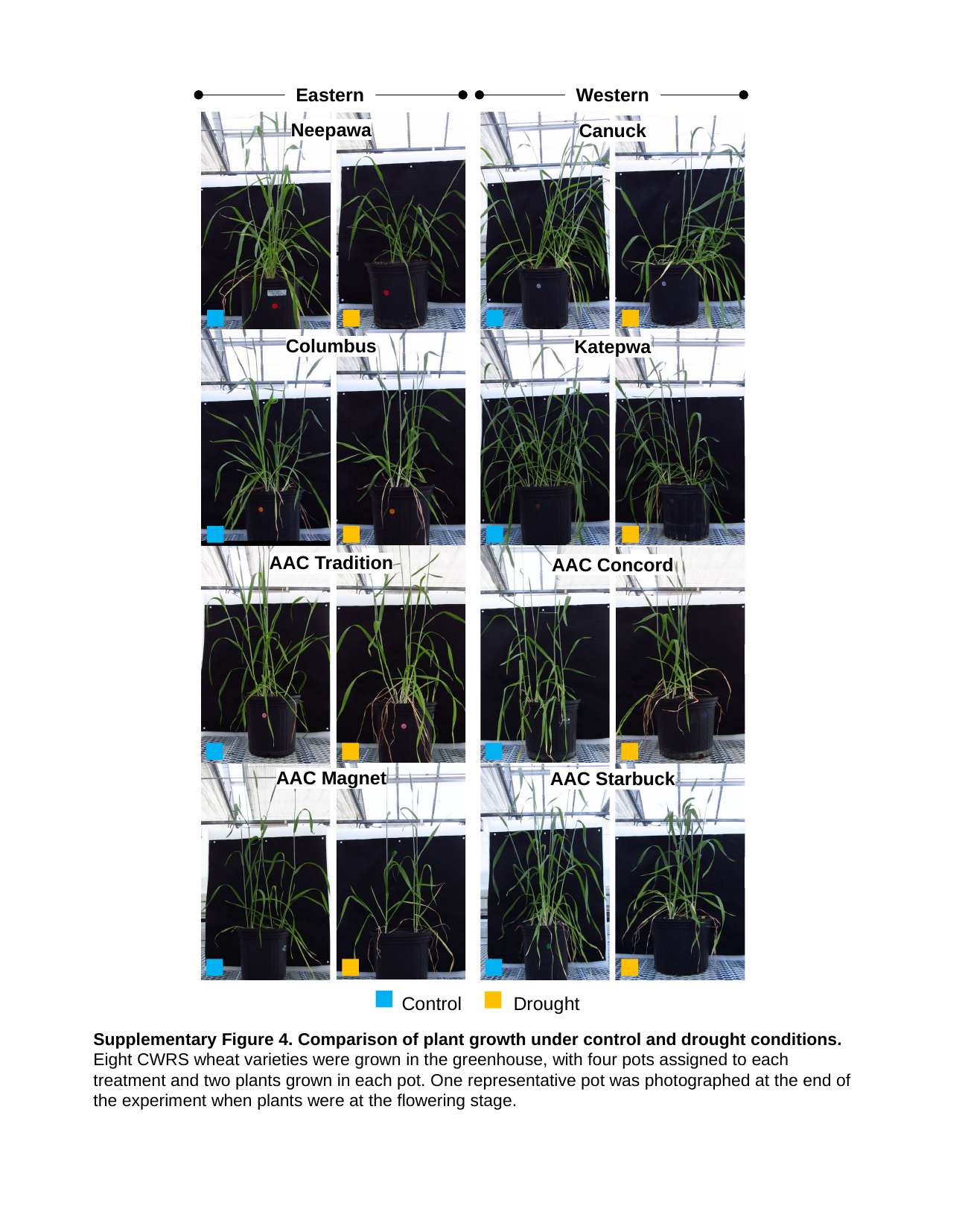

Eastern
Western
Neepawa
Canuck
Columbus
Katepwa
AAC Tradition
AAC Concord
AAC Magnet
AAC Starbuck
Control	Drought
Supplementary Figure 4. Comparison of plant growth under control and drought conditions. Eight CWRS wheat varieties were grown in the greenhouse, with four pots assigned to each treatment and two plants grown in each pot. One representative pot was photographed at the end of the experiment when plants were at the flowering stage.

## Slide 5
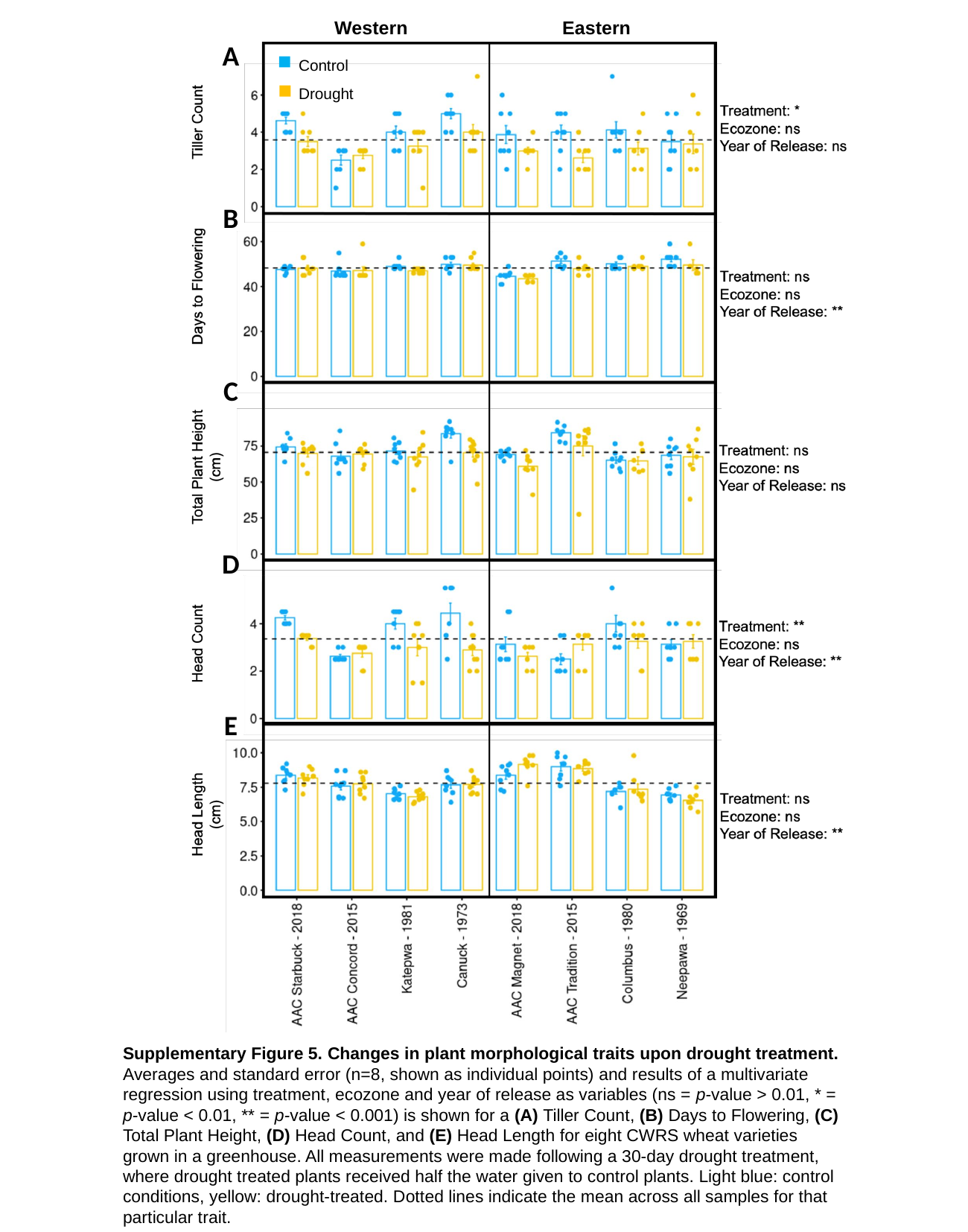

Western
Eastern
A
Control
Drought
B
C
D
E
Supplementary Figure 5. Changes in plant morphological traits upon drought treatment. Averages and standard error (n=8, shown as individual points) and results of a multivariate regression using treatment, ecozone and year of release as variables (ns = p-value > 0.01, * = p-value < 0.01, ** = p-value < 0.001) is shown for a (A) Tiller Count, (B) Days to Flowering, (C) Total Plant Height, (D) Head Count, and (E) Head Length for eight CWRS wheat varieties grown in a greenhouse. All measurements were made following a 30-day drought treatment, where drought treated plants received half the water given to control plants. Light blue: control conditions, yellow: drought-treated. Dotted lines indicate the mean across all samples for that particular trait.

## Slide 6
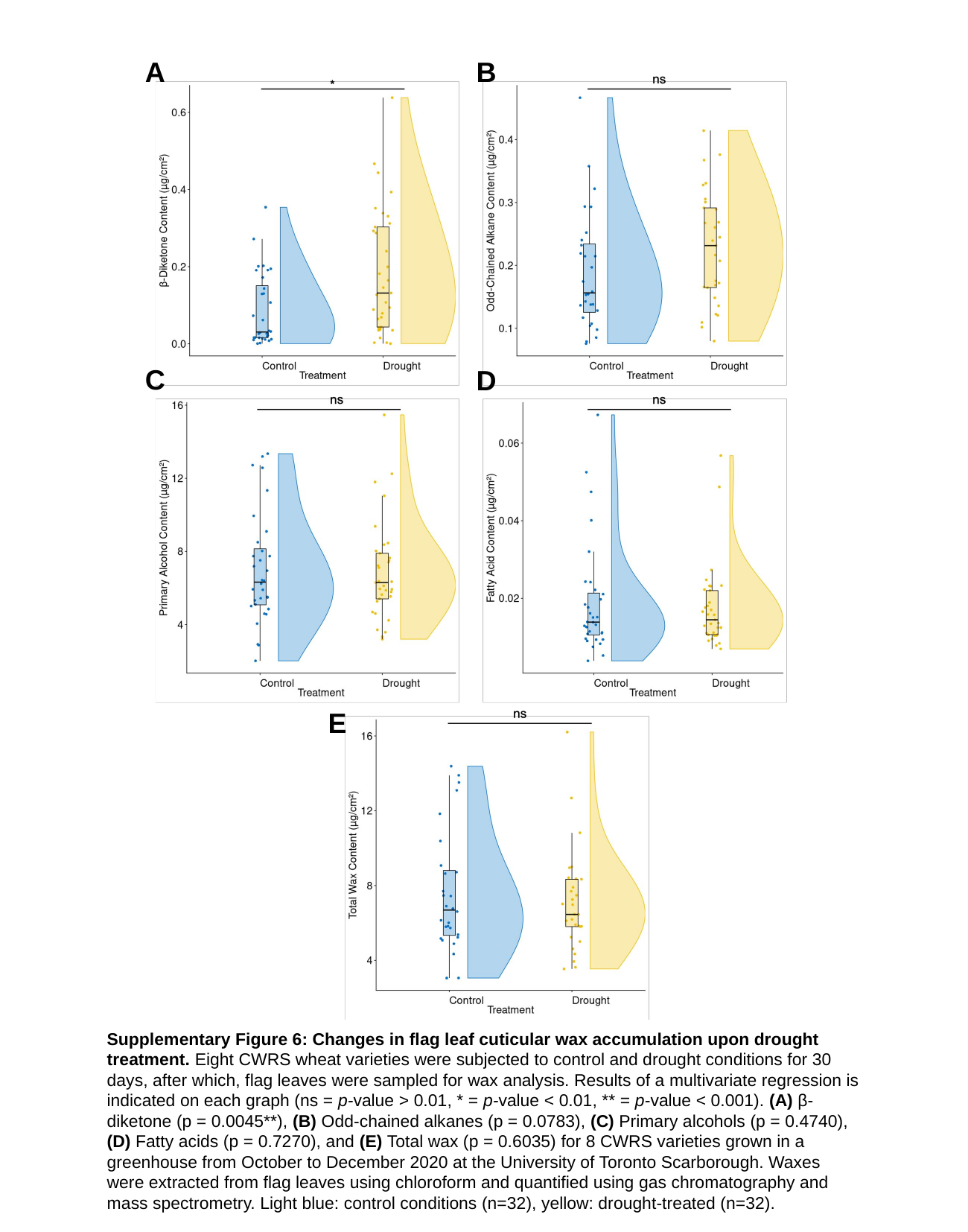

A
B
C
D
E
Supplementary Figure 6: Changes in flag leaf cuticular wax accumulation upon drought treatment. Eight CWRS wheat varieties were subjected to control and drought conditions for 30 days, after which, flag leaves were sampled for wax analysis. Results of a multivariate regression is indicated on each graph (ns = p-value > 0.01, * = p-value < 0.01, ** = p-value < 0.001). (A) β-diketone (p = 0.0045**), (B) Odd-chained alkanes (p = 0.0783), (C) Primary alcohols (p = 0.4740), (D) Fatty acids (p = 0.7270), and (E) Total wax (p = 0.6035) for 8 CWRS varieties grown in a greenhouse from October to December 2020 at the University of Toronto Scarborough. Waxes were extracted from flag leaves using chloroform and quantified using gas chromatography and mass spectrometry. Light blue: control conditions (n=32), yellow: drought-treated (n=32).

## Slide 7
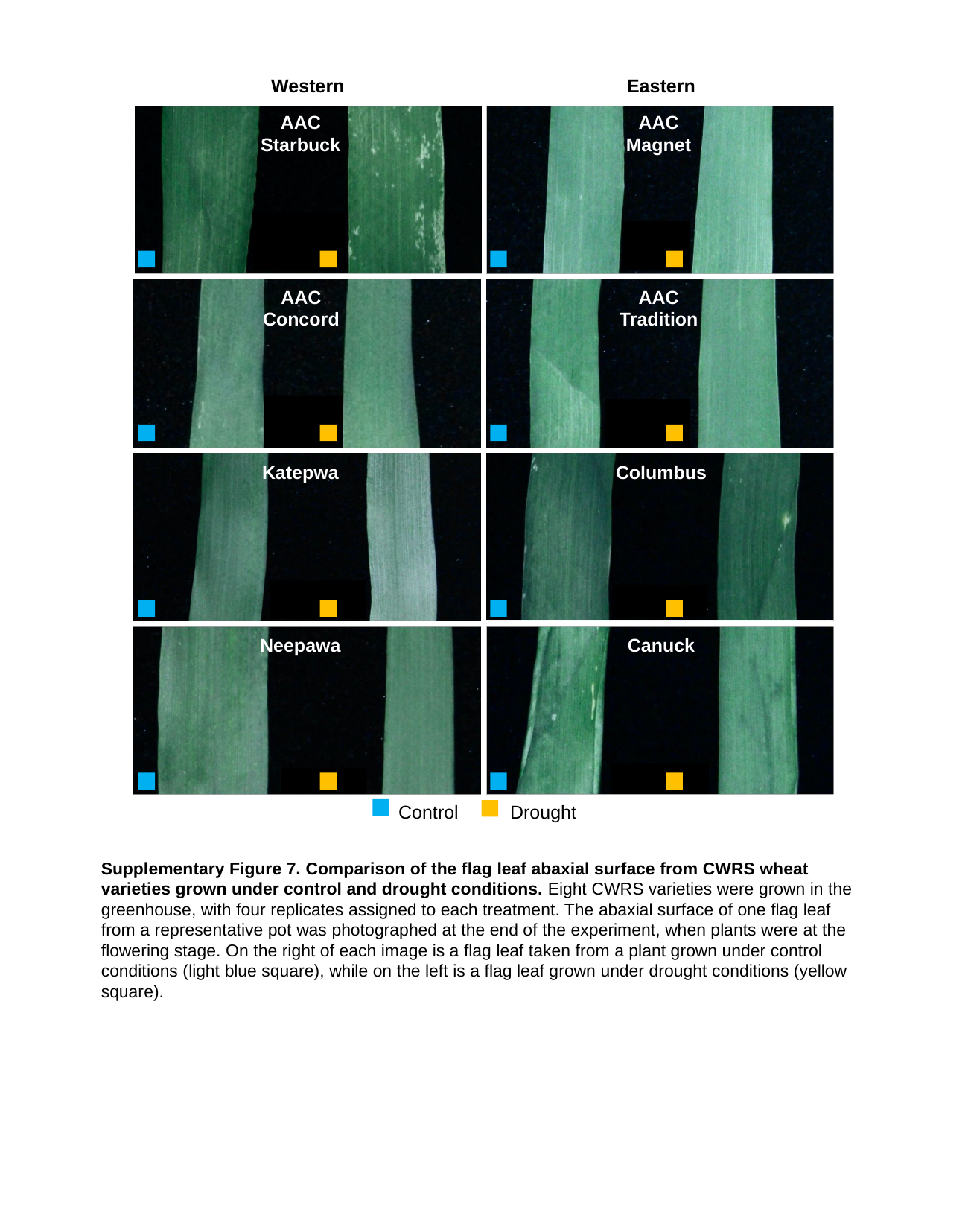

Western
Eastern
AAC
Starbuck
AAC
Magnet
AAC
Tradition
AAC
Concord
Katepwa
Columbus
Neepawa
Canuck
Control	Drought
Supplementary Figure 7. Comparison of the flag leaf abaxial surface from CWRS wheat varieties grown under control and drought conditions. Eight CWRS varieties were grown in the greenhouse, with four replicates assigned to each treatment. The abaxial surface of one flag leaf from a representative pot was photographed at the end of the experiment, when plants were at the flowering stage. On the right of each image is a flag leaf taken from a plant grown under control conditions (light blue square), while on the left is a flag leaf grown under drought conditions (yellow square).

## Slide 8
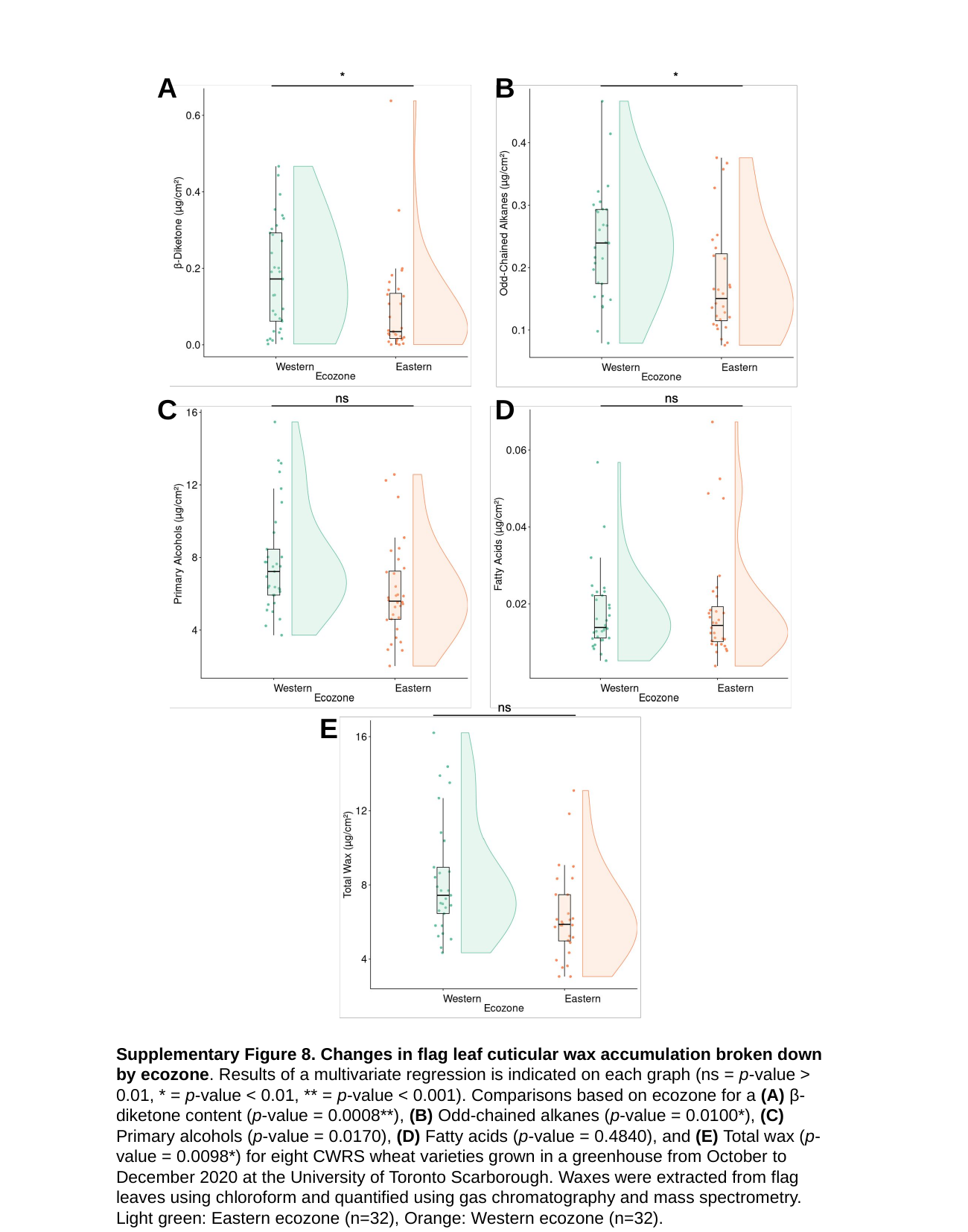

A
B
C
D
E
Supplementary Figure 8. Changes in flag leaf cuticular wax accumulation broken down by ecozone. Results of a multivariate regression is indicated on each graph (ns = p-value > 0.01, * = p-value < 0.01, ** = p-value < 0.001). Comparisons based on ecozone for a (A) β-diketone content (p-value = 0.0008**), (B) Odd-chained alkanes (p-value = 0.0100*), (C) Primary alcohols (p-value = 0.0170), (D) Fatty acids (p-value = 0.4840), and (E) Total wax (p-value = 0.0098*) for eight CWRS wheat varieties grown in a greenhouse from October to December 2020 at the University of Toronto Scarborough. Waxes were extracted from flag leaves using chloroform and quantified using gas chromatography and mass spectrometry. Light green: Eastern ecozone (n=32), Orange: Western ecozone (n=32).

## Slide 9
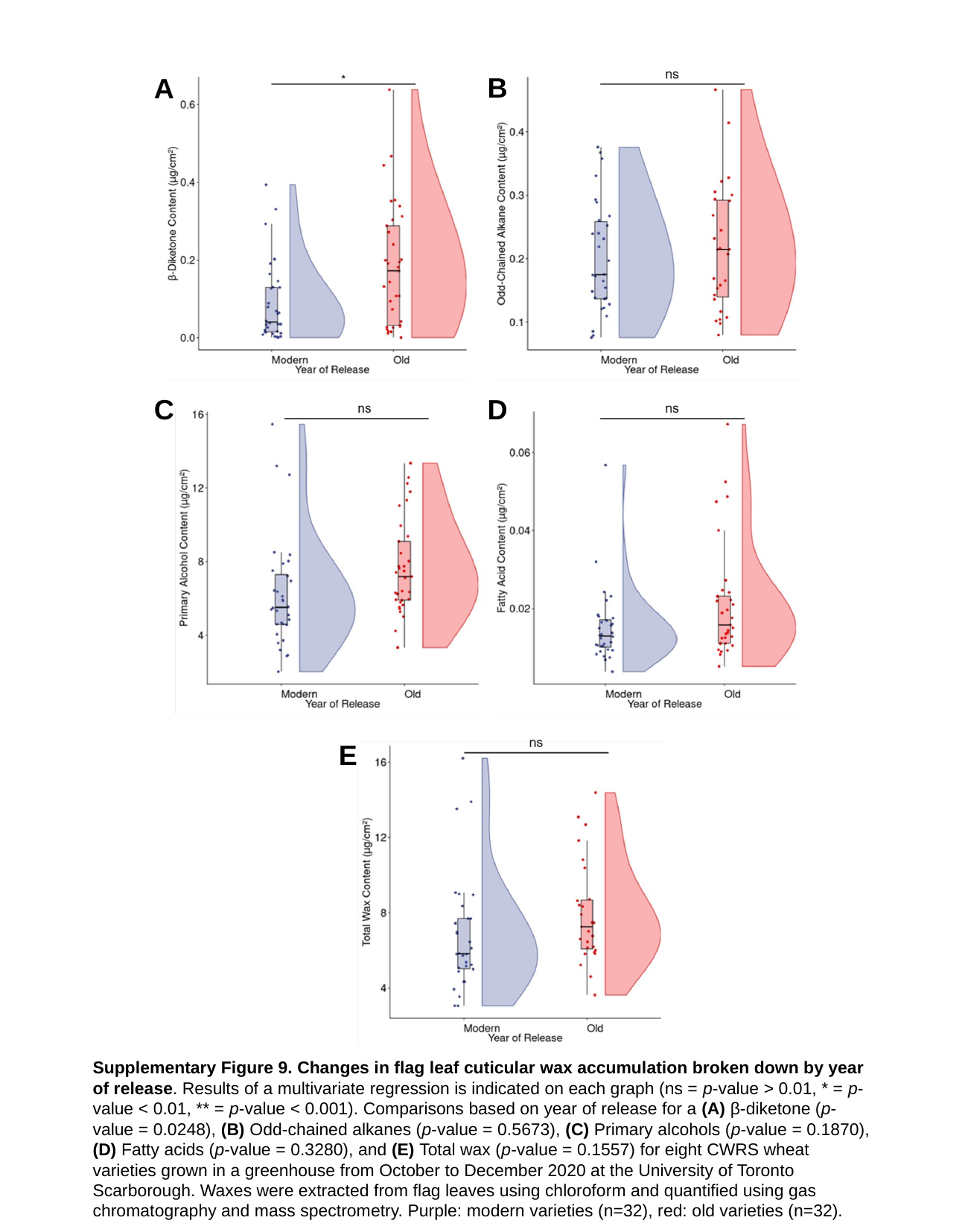

B
A
C
D
E
Supplementary Figure 9. Changes in flag leaf cuticular wax accumulation broken down by year of release. Results of a multivariate regression is indicated on each graph (ns = p-value > 0.01, * = p-value < 0.01, ** = p-value < 0.001). Comparisons based on year of release for a (A) β-diketone (p-value = 0.0248), (B) Odd-chained alkanes (p-value = 0.5673), (C) Primary alcohols (p-value = 0.1870), (D) Fatty acids (p-value = 0.3280), and (E) Total wax (p-value = 0.1557) for eight CWRS wheat varieties grown in a greenhouse from October to December 2020 at the University of Toronto Scarborough. Waxes were extracted from flag leaves using chloroform and quantified using gas chromatography and mass spectrometry. Purple: modern varieties (n=32), red: old varieties (n=32).

## Slide 10
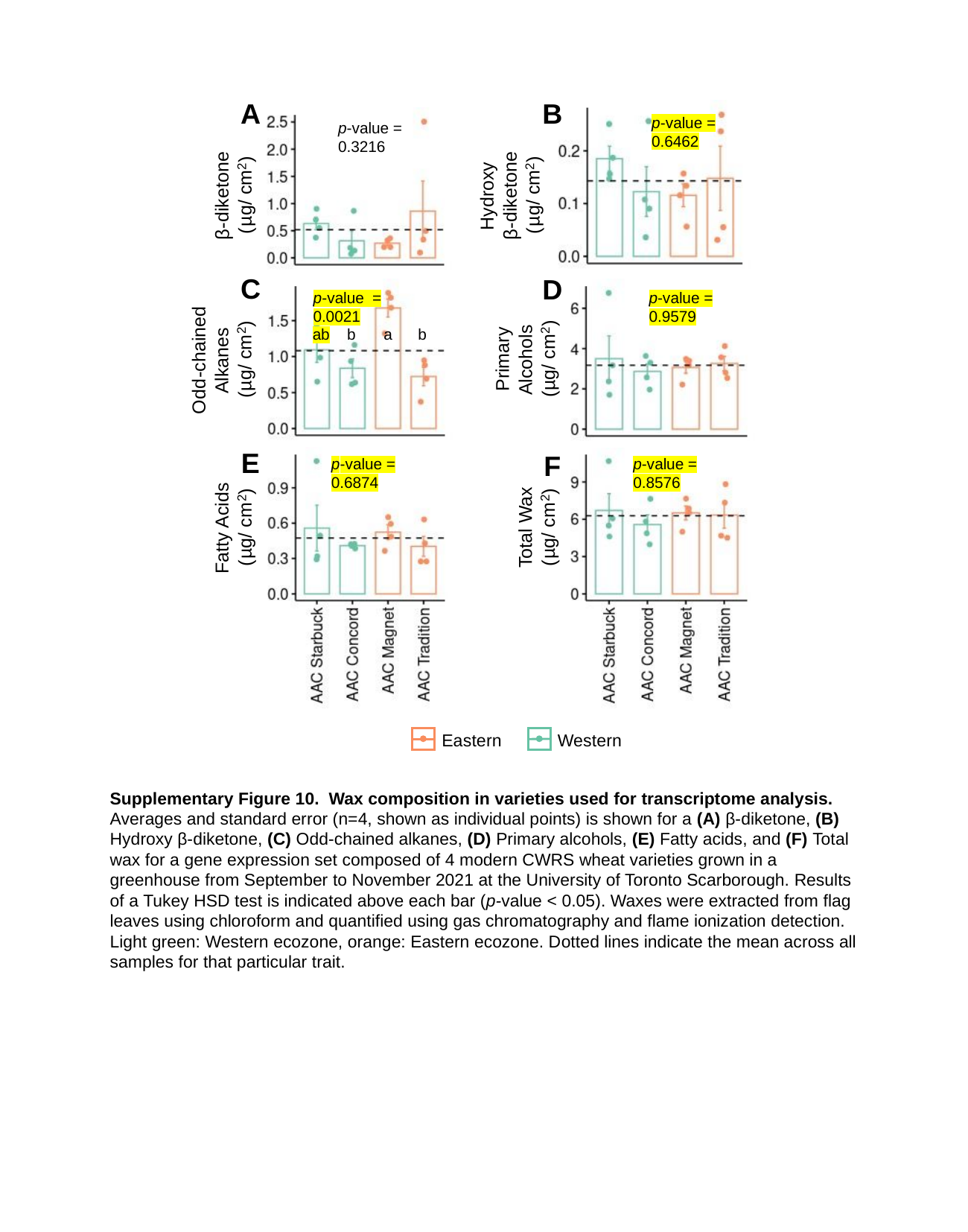

A
B
p-value = 0.6462
p-value = 0.3216
)
)
2
2
diketone
diketone
Hydroxy
µg/ cm
µg/ cm
-
-
(
(
β
β
p-value = 0.0021
p-value = 0.9579
)
)
2
2
ab
b
a
b
chained
Primary
Alcohols
Alkanes
µg/ cm
µg/ cm
-
(
(
Odd
p-value = 0.6874
p-value = 0.8576
)
)
2
2
Total Wax
Fatty Acids
µg/ cm
µg/ cm
(
(
Eastern
Western
C
D
E
F
Supplementary Figure 10. Wax composition in varieties used for transcriptome analysis. Averages and standard error (n=4, shown as individual points) is shown for a (A) β-diketone, (B) Hydroxy β-diketone, (C) Odd-chained alkanes, (D) Primary alcohols, (E) Fatty acids, and (F) Total wax for a gene expression set composed of 4 modern CWRS wheat varieties grown in a greenhouse from September to November 2021 at the University of Toronto Scarborough. Results of a Tukey HSD test is indicated above each bar (p-value < 0.05). Waxes were extracted from flag leaves using chloroform and quantified using gas chromatography and flame ionization detection. Light green: Western ecozone, orange: Eastern ecozone. Dotted lines indicate the mean across all samples for that particular trait.
